# Development of a Fertility Restorer for *inap* CMS (*Isatis indigotica*) *Brassica napus* Through Genetic Introgression of One Alien Addition

**DOI:** 10.3389/fpls.2019.00257

**Published:** 2019-03-05

**Authors:** Pengfei Li, Lei Kang, Aifan Wang, Cheng Cui, Liangcai Jiang, Shizhen Guo, Xianhong Ge, Zaiyun Li

**Affiliations:** ^1^National Key Laboratory of Crop Genetic Improvement, National Center of Oil Crop Improvement (Wuhan), College of Plant Science and Technology, Huazhong Agricultural University, Wuhan, China; ^2^Crop Research Institute, Sichuan Academy of Agricultural Sciences, Chengdu, China

**Keywords:** *Brassica napus*, cytoplasmic male sterility, restorer of fertility, nuclear-mitochondrial incompatibility, monosomic alien addition line

## Abstract

Novel *Brassica napus* cytoplasmic male sterility (CMS) with carpelloid stamens (*inap* CMS) was produced by intertribal somatic hybridization with *Isatis indigotica* (Chinese woad), but its *RF* (restorer of fertility) gene(s) existed in one particular woad chromosome that was carried by one fertile monosomic alien addition line (MAAL) of rapeseed. Herein, the selfed progenies of this MAAL were extensively selected and analyzed to screen the rapeseed-type plants (2*n* = 38) with good male fertility and to produce their doubled haploid (DH) lines by microspore culture. From the investigation of fertility restoration in the F_1_ hybrids with *inap* CMS, one DH line (RF 39) was identified to adequately restore male fertility and likely carried one dominant *RF* gene. Specifically, this restorer produced brown pollen grains, similar to the woad and the MAAL, suggesting that this trait is closely linked with the *RF* gene(s) and serves as one phenotypic marker for the restorer. This restorer contained 38 chromosomes of rapeseed and no intact chromosomes of woad, but some DNA fragments of woad origin were detected at low frequency. This restorer was much improved for pollen and seed fertility and for low glucosinolate content. The successful breeding of the restorer for *inap* CMS rendered this new pollination control system feasible for rapeseed hybrid production.

## Introduction

Male sterility in plants can be caused either by mitochondrial genes coupled with nuclear genes or by nuclear genes alone, known as cytoplasmic male sterility (CMS) and genic male sterility (GMS), respectively (Chen and Liu, 2014). As the CMS trait is maternally inherited and easy to maintain (Hanson and Bentolila, 2004), a CMS fertility restoration system has been widely used as an excellent pollination control system to facilitate hybrid seed production for many crops, particularly for predominantly self-fertilized crops, including oilseed rape (*Brassica napus* L.), thus allowing breeders to realize yield gains resulting from the expression of heterosis.

Depending on the origin of the sterility inducing the cytoplasm, two types of CMS lines are usually distinguished: autoplasmy CMS, which arises spontaneously due to mutation in the genome of one species, and alloplasmy CMS, which occurs following cytoplasmic substitutions between two species due to nuclear-mitochondrial incompatibility (Prakash et al., 2009). The most popular autoplasmy CMS of rapeseed (*B. napus* L.) is *polima* CMS, which has been widely used worldwide during past decades (Fu et al., 1990) and is still dominant in hybrid seed production in China. As *Brassica* coenospecies provide rich and diverse mitochondrial genomes (Pradhan et al., 1992), alloplasmy CMS lines have been developed following backcrossing of either sexually synthesized allopolyploids or somatic hybrids between wild and crop species to diversify the CMS sources for breeding (For review see Prakash et al., 2009). Expectedly, CMS lines from sexual hybridizations maintain unaltered organellar genomes from cytoplasmic donor species because of exclusive maternal inheritance, but those from somatic hybrids likely contain a different cytoplasmic constitution because various possible combinations of mitochondrial and chloroplast genomes have been revealed in different CMS lines due to frequent organelle assortment and intergenomic mitochondrial recombinants in Brassicaceae (Prakash et al., 2009). The *Ogura* CMS found in a wild population of radish (*Raphanus sativus*) (Ogura, 1968) was introduced into *Brassica* species through interspecific crosses (Heyn, 1976; Bannerot et al., 1977) and subsequent protoplast fusion (Pelletier et al., 1983). Since then, a large number of alloplasmics have been produced, mostly for *B. napus* and *B. juncea* (Prakash et al., 2009). However, only *Ogura*-INRA *B. napus* CMS (Friedt et al., 2018) and *Mori B. juncea* CMS, produced from somatic fusion with *Moricandia arvensis* (Singh, 2008), were successfully exploited for commercial seed production on a large scale after systematic improvement of developmental abnormalities, including leaf chlorosis (Pelletier et al., 1983; Kirti et al., 1998).

The development of a fertility restorer for alloplasmic CMS is a challenging task because its fertility restoration gene(s) has been shown to exist in one particular chromosome of the nuclear genome of the cytoplasm donor species (Prakash et al., 1998; Bang et al., 2002; Leino et al., 2004; Budahn et al., 2008; Wei et al., 2010; Kang et al., 2014), owing to the co-evolutionary relationship between the nucleus and cytoplasm (Fujii et al., 2011; Chen and Liu, 2014). The breeding process for restorer lines involves the introgression of the alien chromosomal segments carrying the *restorer of fertility* (*RF*, or restorer) gene(s) and selection of cytologically stable lines with the same chromosome number as the recipient species and with good agronomic value. Theoretically, these *Rf* genes could be introgressed into the recipient species by taking advantage of non-homologous allosyndetic recombination in backcrossing or selfing generations of sexual or somatic hybrids. The genetic improvement of the introgression lines, which meet the breeding requirements, is demonstrated to be very time and effort consuming, especially when a large amount of alien element is introduced and associated with linkage drag, leading to deleterious characters, such as poor agronomic performance (Delourme et al., 1998; Giancola et al., 2003).

**FIGURE 1:**
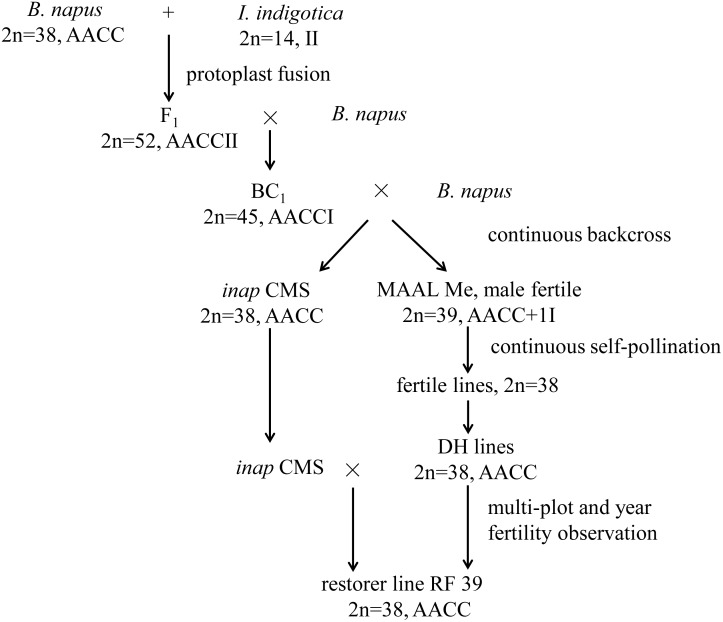
The breeding procedure of restorer line for *inap* CMS.

In our previous study, the intertribal somatic hybrids (2*n* = 52, AACCII) between *B. napus* (2*n* = 38, AACC) and *Isatis indigotica* Fort. (Chinese woad; 2*n* = 14, II) of the Isatideae tribe within the Brassicaceae family were obtained (Du et al., 2009) and backcrossed continuously to *B. napus*, resulting in the development of one novel *B. napus* CMS line with carpelloid stamens (named *inap* CMS) (Kang et al., 2017; Figure 1). The *inap* CMS contained the majority of mitochondrial genes from woad and one recombinant of two species. No fertility restorer for *inap* CMS was found among the restorers of other autoplasmic and alloplasmic CMS systems and among a large number of *B. napus* genotypes with extensive resources. Alternatively, a complete set of monosomic alien addition lines (MAALs) of *B. napus*, with one of seven woad chromosomes and with the same cytoplasm as *inap* CMS (artificially designated as Ma-Mg), was also established and characterized among the backcrossing progenies of the somatic hybrids (Figure 1; Kang et al., 2014). Only MAAL Me produced normal anthers with a brown color, a trait of woad origin, which contained viable pollen grains, but six other MAALs had carpelloid stamens, as did *inap* CMS. This suggested that this woad chromosome harbored the gene(s) that should rectify the feminized development of the stamens and restore the male fertility of *inap* CMS. Then the restorer line with the *B. napus*-like chromosome complement could probably be selected among the selfed progenies of Me when the chromosomal segment, related to floral development, was introgressed via certain pathways. In the present study, extensive selections and investigations of phenotype, male fertility, cytology, and genomic composition were conducted for the progenies of Me with the aims to breed a restorer line for *inap* CMS and to improve its fertility and agronomic performance.

## Materials and Methods

### Plant Materials

As only one *B. napus*-*I. indigotica* MAAL (Me) among all seven MAALs (in the presence of the sterility-inducing cytoplasm of *inap* CMS) had normal flowers with brown anthers, as did *I. indigotica* (Kang et al., 2014), its progenies by self-pollination from three generations were screened for plants with high pollen fertility and with the same 2*n* = 38 as *B. napus* during 2012–2014 in Wuhan, Hubei Province and Xining, Qinghai Province. Individuals with normal stamens were selected for microspore culture in the spring of 2014. Subsequently, doubled haploid (DH) plants with normal stamens and high pollen fertility were chosen for testcrossing with *inap* CMS in the spring of 2015. Then, DH lines and their F_1_ hybrids were planted together with the maintainer line *B. napus* cv. Huashuang 3 (H3) with double-low quality and the *inap* CMS line in Wuhan, Chengdu, Changsha (winter-type growing regions) and Xining (spring-type region) for fertility evaluation from 2015 to 2018. One restorer selected among DH lines was crossed with thirty rapeseed cultivars of wide origins to improve its fertility and agronomic performance.

### Microspore Culture

To obtain pure lines of the selfed progenies from Me, a microspore culture regenerative technique was performed in March 2014 according to Lichter (1982) with minor modifications. Flower buds, all with a length of 2.5–4 mm, were picked out from the main inflorescence of fertile individuals between 8:00 and 10:00 AM to extract microspores. The isolated microspores were resuspended in 10 ml of NLN-16 medium with the addition of 60 mg l^-1^ colchicine at 32°C in darkness for 53 h and then incubated at 25°C in darkness until cotyledonous embryoids appeared. The plants generated from microspore culture were transplanted in the field in October 2014.

### Plant Phenotype and Seed Quality

The maintainer, restorer, and F_1_ hybrid were compared for the following parameters: flower organs, including pistil length, flower diameter, sepal length, long stamen length, short stamen length, petal length, and petal width, by measuring 10 fully opened flowers on the same sunny day for each line. Their flowering and maturing times were counted from planting in 2016–2017 and 2017–2018 in Wuhan. At maturity, 10 representative plants of these lines were observed for agronomic traits. For the fatty acid compositions, mature seeds were measured by using near infra-red spectroscopy (NIRS) (Foss NIRS Systems 5000, KWS UK). The glucosinolate (GSL) profiles of the maintainer, restorer, and woad with two replicates were analyzed by the Institute of Oil Crops Research, Chinese Academy of Agricultural Sciences, Wuhan, with high-performance liquid chromatography (HPLC, YLSB077, 1200).

### Cytology and Pollen Viability Analysis

To observe the chromosome numbers and meiosis of fertility restored plants and testcross progenies, young flower buds were collected in the morning. For mitosis, the ovaries were treated with 2 mM 8-hydroxyquinoline solution for 3 h at room temperature before being fixed in Carnoy’s solution I (3:1 ethanol:glacial acetic acid, v/v) and stored at -20°C for further chromosome counting of somatic cells. For meiosis, the young flower buds were fixed directly in Carnoy’s solution I and stored at -20°C. Cytogenetic observations were carried out as described by Li et al. (1995). Pollen grains of each plant were stained with acetocarmine (1%) and more than 300 were counted; the percentage of stainable pollen grains was calculated to evaluate pollen fertility.

### Probe Labeling and GISH (Genomic *in situ* Hybridization) Analysis

Total genomic DNA was extracted from young leaves as described by Dellaporta et al. (1983). Genomic DNA of *I. indigotica* was labeled with Bio-11-dUTP (Fermentas) by random priming using the BioPrime DNA Labeling System kit (Invitrogen, Life Technologies). Total genomic DNA of *B. napus* was boiled twice, 15 min each time, to obtain DNA fragments of 100–500 bp that were used as blocks.

Chromosomes for GISH were prepared as described by Ge and Li (2007), and *in situ* hybridization was carried out according to Zhu et al. (2016). Pictures were taken using a fluorescence microscope (Axio Scope A1, Zeiss, Germany) with a CCD camera. Images were processed by Adobe Photoshop CS5 to adjust contrast and brightness.

### Genomic Analysis

To identify the *I. indigotica* DNA segments introgressed into the restorer lines, 21 SSR markers specific for MAAL Me identified by Kang et al. (2014) and one centromeric marker were used. PCR amplifications were performed in a volume of 10 μl containing 20 ng genomic DNA, 1×*Taq* buffer, 2 mM MgCl_2_, 0.2 U *Taq* DNA polymerase (Fermentas), 2 mM dNTPs (Fermentas), 5 μM forward and reverse primers. The PCR program was as follows: 94°C for 5 min; 10 cycles at 94°C for 30 s, 60°C for 30 s, 72°C for 45 s, with a 0.5°C decrease in annealing temperature in each cycle; 30 cycles at 94°C for 30 s, 55°C for 30 s, 72°C for 45 s, and a final extension at 72°C for 10 min. PCR products were separated on 1% agarose gels.

AFLP analysis was performed on the maintainer (Huashuang 3), restorer, *inap* CMS, woad, additional line Me, and F_1_ plants following the procedures of Vos et al. (1995), with slight modifications. Total purified genomic DNA (50 ng) was digested by the restriction endonucleases *Eco*RI and *Mse*I 6 h at 37°C. Inactivation was carried out at 65°C for 1 h, followed by ligation to *Eco*RI and *Mse*I adapters at 4°C overnight. The resulting ligation products were then amplified by pre-selective PCR with primers matching the adapters. The pre-selective PCR products were used as templates for selective PCR as follows: 94°C for 3 min; 13 cycles at 94°C for 30 s, 65°C for 45 s, 72°C for 1 min, with a 0.7°C decrease in annealing temperature in each cycle; 26 cycles at 94°C for 30 s, 56°C for 45 s, 72°C for 1 min, and a final extension at 72°C for 5 min. Amplification products were separated on 6% denaturing polyacrylamide gels, and photographs were taken.

### Statistical Data Analysis

The statistical significance of the data was calculated, including a *t*-test, an *LSD* test, and a χ^2^ test, using R statistical software.

## Results

### Development of a Restorer for *inap* CMS

Theoretically, MAAL Me, with the male sterility-inducing cytoplasm, should produce three types of progenies after selfing pollination: monosomic and disomic additions, both with small anthers containing viable brown pollen grains, and *B. napus* plants with carpelloid stamens, if the additional chromosome remained intact. These three types of progeny plants were easy to distinguish from their specific floral phenotypes but were not targeted to select the restorer. As the flowers of MAAL Me plants had anthers with a much smaller size than the *B. napus* recipient cultivar H3 (Kang et al., 2014), artificial supplementary pollination was performed to obtain more seeds. Thousands of seeds were harvested but some were found to have germinated in pods prior to maturity, probably resulting from development disturbance by alien chromosome or cytoplasm. Subsequently, these seeds were sown during three growing seasons in Wuhan and Xining to screen the individuals with male fertility and 2*n* = 38. In total, 60 plants were primarily selected by their larger flowers and larger anthers, compared with MAAL Me, but showed variable seed-sets from low to high, likely because they had shorter and thinner stamens with low pollen fertility compared to the maintainer line (H3). Certain parts of the seeds from these plants germinated in pods.

**FIGURE 2:**
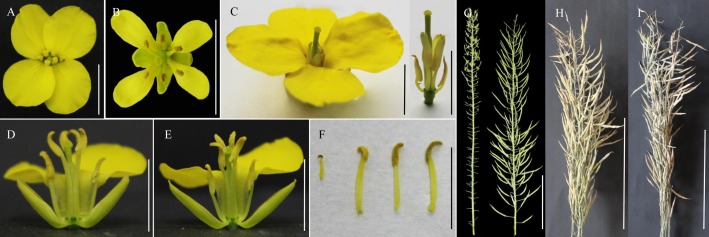
Flowers and inflorescences of *inap* CMS and restorer (RF 39). **(A)** One flower of the *B. napus* maintainer with yellow anthers and pollen grains. **(B)** One flower of *I. indigotica* with small anthers containing brown pollen grains. **(C)** One flower of *inap* CMS with carpelloid anthers (left) and the same flower with only carpelloid anthers and pistil (right). With the increase of backcrossing generations to the maintainer, the size of the carpelloid anthers reduces and their feminized structure degenerates. **(D,E)** One flower of RF 39 and F_1_ hybrid with some sepals and petals removed, respectively, to show the six well developed anthers. Notice the brownish yellow color of the pollen grains. **(F)** The comparison of the stamens from *I. indigotica*, *B. napus*, RF 39 and F_1_ (left to right) with the focus on the different colors of the pollen grains. Bars: **(A–F)** 1 cm. **(G)** Sterile and fertile inflorescences of the hybrid plants after *inap* CMS and RF 39 are pollinated by the maintainer as male and female, respectively. **(H,I)** Mature bagged inflorescences of RF 39 and F_1_ hybrid with good seed-sets, respectively. Bars: **(G–I)** 20 cm.

To stabilize these fertile progenies of the potential restorer, microspore culture was performed for 60 screened strains and 625 plants were obtained; in total, 192 DH plants, originating from all of the parental lines, were established and investigated for male development. While most of these DH plants (140/192) exhibited carpelloid stamens, 52 developed flowers with different numbers of stamens, mostly with large tetradynamous stamens but 2 tiny stamens, and only a few with 6 well-developed stamens. Although the stamens of these DH plants produced some stainable pollen grains, they contained obviously fewer pollen grains than *B. napus* and also showed poorer dehiscence. Therefore, the seed sets varied greatly for these plants. F_1_ hybrids between 52 DH plants and the *inap* CMS line were produced in spring 2015 and were observed for fertility restoration in four locations (Wuhan, Chengdu, Changsha and Xining) from 2015 to 2018. These 52 hybrids showed different degrees of male fertility by producing flowers with variable numbers of anthers (1–6) or a mixture of normal and carpelloid stamens. Only hybrids with DH plant No. 39 developed six normal stamens and consistently had good seed sets at four locations over the years. Plant No. 39 and its hybrids had high pollen stainability (91.04 and 91.52%, respectively) (Table 1), and produced long siliques with good seed sets after being bagged for self-pollination (Figure 2H,I). However, the seed number per pod was 11.8 for Plant No. 39 and 16.4 for the hybrid, lower than 19.5 for the maintainer H3. The phenomenon of the seed germination in pods prior to maturity was not observed for Plant No. 39 and its hybrid. Therefore, DH Line 39 had the ability to rectify the feminized development of the stamens in *inap* CMS and to restore male fertility in their F_1_ hybrids, which served as a restorer line and was designated as RF 39.

**Table 1:** Flower traits and pollen fertility of the *B. napus* maintainer (H3), RF 39 and F_1_ hybrid.

Line	Pistil length (cm)	Flower diameter (cm)	Sepal length (cm)	Sepal width (cm)
H3	0.79^a^ ± 0.03	2.10^a^ ± 0.11	0.75^a^ ± 0.04	0.21^a^ ± 0.02
RF 39	0.77^a^ ± 0.07	2.00^a^ ± 0.13	0.74^a^ ± 0.04	0.19^a^ ± 0.02
F1	0.81^a^ ± 0.03	2.25^b^± 0.06	0.81^b^ ± 0.03	0.24^b^ ± 0.01

**Petal length (cm)**	**Petal width (cm)**	**Long stamens length (cm)**	**Short stamens length (cm)**	**Pollen viability (%)**
0.92^a^ ± 0.06	0.94^a^ ± 0.06	1.02^a^ ± 0.02	0.80^a^ ± 0.04	94.25^a^ ± 1.96
0.91^a^ ± 0.06	0.91^a^ ± 0.08	0.95^b^ ± 0.06	0.73^b^ ± 0.07	91.04^b^ ± 2.77
1.00^b^ ± 0.03	1.01^b^ ± 0.04	0.93^b^ ± 0.06	0.69^b^ ± 0.12	91.52^b^ ± 2.04

*For each parameter, means in the columns followed by the same letters are not significantly different at 0.05 probability level by t-test.*

### Phenotype of Restorer RF 39

With respect to the floral organs, RF 39 with the same cytoplasm as *inap* CMS-produced flowers with a slightly smaller size than those of the maintainer (Table 1), but its flowers were distinguishable by some curled petals, instead of the very smooth petals observed in the maintainer (Figure 2A,D). The four long stamens and two short stamens were shorter than those of the maintainer, were less plump and released few pollen grains due to poorer dehiscence (Figure 2D). In particular, the pollen grains of RF 39 and F_1_ hybrids were brown, similarly to those of Chinese woad and MAAL Me, while those of the maintainer were yellow (Figure 2A,B,D–F). The brown pollen color trait, which was associated with anther development on the woad chromosome, was dominant over the yellow pollen of *B. napus* in the somatic hybrids (Du et al., 2009), MAAL Me (Kang et al., 2014) and the introgressant restorer (present study). This revealed that the gene (s) responsible for the brown pollen color was (were) closely linked with the *RF* gene(s) and provided one phenotypic marker for the restorer line.

The whole plant architecture of RF 39 with the sterility-inducing cytoplasm was similar to that of the *inap* CMS but it was smaller than that of the maintainer (data unshown), except for the different floral organs and male fertilities (Figure 2A,C,D). It showed an obviously poorer seed set under selfing or open pollination than the maintainer, as its pods had fewer seeds (11.8 vs. 19.5). This most likely resulted from the negative effect of the sterility-inducing cytoplasm, as its hybrid with the maintainer as the female showed normal seed fertility (Figure 2G). Notably, the plants of RF 39 flowered almost 2 weeks later than the maintainer Huashuang 3 when sown in the autumn in Wuhan, similarly to the *inap* CMS (Kang et al., 2017). The delayed development of *inap* CMS and RF 39 might result from the detrimental cytoplasmic effect related to aberrant mitochondrial functions.

### Seed Quality of Restorer RF 39

The seeds of *I. indigotica* had much higher glucosinolate (GSL) content (102.95 μmol/g) than those of the *B. napus* maintainer (14.07 μmol/g) and also quite different compositions of GSLs (Table 2), as they contained only three kinds of GSLs (sinigrin, SIN; progoitrin, PRO; gluconapin, NAP), and SIN was the predominant component (77%). In the seeds of the maintainer with a profile of 11 GSLs, 4-hydroxyglucobrassicin (4HGBS) (40%) and gluconasturtiin (GST) (29%) were dominant, but the others appeared at very low percentages. The major SIN in the woad was absent in the maintainer, and the main 4HGBS and GST in the maintainer were not detected in the woad. The seeds of RF 39 had a higher GSL content (60.51 μmol/g) than those of the maintainer and mainly contained NAP (56%), PRO (16%), and 4HGBS (11%), while their types of GSLs were very similar to each other. Intriguingly, NAP (34.15 μmol/g, 56%) was the overwhelming component among 13 GSLs of RF 39, while its content (1.22 μmol/g, 9%) was lower in the maintainer, but it was the third most common (9.94 μmol/g, 10%) in the woad. The rate of PRO in RF 39 (16%) was higher than that in the maintainer (6%) and the woad (13%), but the rate of 4HGBS (11%) was much lower than that in the maintainer (40%). RF 39 did not produce the predominant SIN in the woad, in contrast to the maintainer. Therefore, compared to the maintainer, the increase in the GSL content in RF 39 was largely attributable to the enhanced contents of NAP (34.15 vs. 1.22) and PRO (9.56 vs. 0.9).

**Table 2:** Glucosinolate (GSL) content (μmol/g) in the seeds of *B. napus* (H3), woad and RF 39.

Lines	PRO	*trans*-PRO	SIN	GRA	GNL	GAL	NAP
H3	0.90^c^	0.81^b^	0	0.07^b^	0.07^a^	0.09^a^	1.22^c^
Woad	13.41^a^	0	79.6^a^	0	0	0	9.94^b^
RF 39	9.56^b^	4.16^a^	0	0.76^a^	0.04^a^	0.09^a^	34.15^a^

**4-HGBS**	**GBN**	**GBE**	**GTR**	**GST**	**4-MGBS**	**NGBS**	**Total GSLs**
5.64^a^	0.39^a^	0.51^a^	0.34^a^	4.02^a^	0	0	14.07^c^
0	0	0	0	0	0	0	102.95^a^
6.92^a^	1.83^a^	1.37^a^	0.41^a^	1.23^b^	0.03^a^	0.01^a^	60.51^b^

*For each parameter, means in the columns followed by the same letters are not significantly different at 0.05 probability level by LSD test according to one-way ANOVA test, with line as variability factor. PRO, progoitrin; trans-PRO, trans-progoitrin; SIN, sinigrin; GRA, glucoraphanin; GNL, gluconapoleiferin; GAL, glucoalyssin; NAP, gluconapin; 4-HGBS, 4-hydroxyglucobrassicin; GBN, glucobrassicanapin; GBE, glucoberteroin; GTR, glucotropaeolin; GST, gluconasturtiin; 4-MGBS, 4-methoxyglucobrassicin; NGBS, neoglucobrassicin.*

However, the fatty acid compositions of RF 39 showed no obvious differences from those of the maintainer, and a low erucic acid content (∼1%) was maintained, but those of the woad included 45% erucic acid.

### Chromosomal and Genomic Complements of Restorer RF 39

**FIGURE 3:**
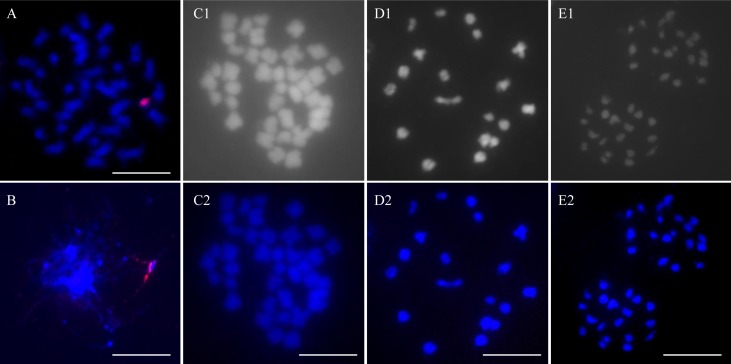
GISH analysis of the alien additional line and restorer. Blue color is from the DAPI staining of chromosomes. Red color is from the signal of the labeled genomic probe of *I. indigotica*. **(A)** One mitotic ovary cell of MAAL Me with one chromosome from *I. indigotica* (red). **(B)** One PMC of MAAL Me at meiotic pachytene with homoeologous association between the additional woad chromosome (red) and the other of rapeseed. **(C1,C2)** One mitotic ovary cell of RF 39 with only 38 unlabeled chromosomes from *B. napus* but no signals of *I. indigotica* on these chromosomes. **(D1,D2)** One diakinesis PMC of RF 39 with 19 bivalents unlabeled by the *I. indigotica* probe. **(E1,E2)** One PMC at anaphase of RF 39 with equal 19:19 segregation and with no labeled chromosomes in each polar group by the *I. indigotica* probe. Arrows indicate alien chromosomes. Bars: 10 μm.

Chromosome number and the genomic complement of RF 39 were determined in mitotic and meiotic cells by conventional and molecular cytogenetic methods. In the somatic hybrids between *B. napus*, *I. indigotica* and MAALs, the chromosomes from *I. indigotica* were characterized by their much smaller sizes than those of *B. napus* (Du et al., 2009; Kang et al., 2014). In MAAL Me, one alien chromosome of woad origin was clearly identified to be of small size by GISH analysis with its labeled genomic DNA as a probe (Figure 3A). In some pollen mother cells at the meiotic zygotene/pachytene stage, this woad chromosome was associated with the other from *B. napus* (Figure 3B), likely indicating homoeologous pairing and exchange, and providing some explanation for the DNA introgressions detected. All of the observed somatic and meiotic cells of RF 39 included only 38 chromosomes, originating from *B. napus*, and no intact chromosome or chromosomal segment from *I. indigotica*, as no obvious signals of the woad probe were even detected on these chromosomes (Figure 3C–E). These chromosomes were paired as 19 bivalents at diakinesis and segregated equally as 19:19 at anaphase I (Figure 3D,E). No laggards occurred during meiosis I and II. Regular meiosis ensured the formation of pollen grains with high viability.

**FIGURE 4:**
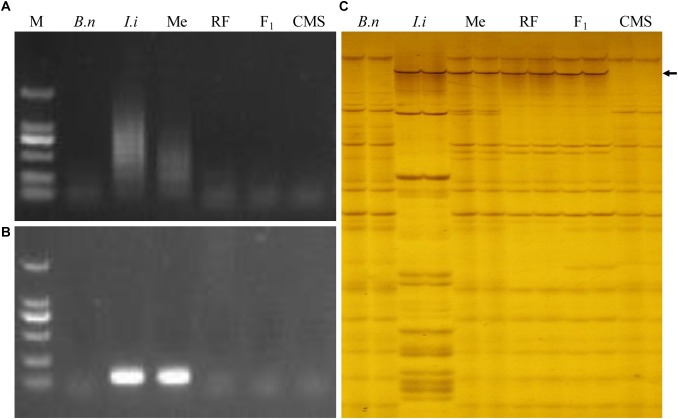
Molecular analysis for genomic complements of the restorer. **(A)** The PCR profile by the woad centromere specific marker with amplified bands only in woad and MAAL Me. **(B)** The woad chromosome specific marker SSR79 amplifies the bands only in woad and MAAL Me. **(C)** AFLP profile from marker EA15/MC6 with amplified bands in woad, MAAL Me, RF 39 and F_1_ (black arrow) but not in rapeseed. *B. n* represents for *B. napus*, *I. i* for *I. indigotica*, Me for MAAL Me, RF for RF 39, F_1_ for hybrid of RF 39 and *inap* CMS, CMS for *inap* CMS. M DL2000.

The woad-specific SSR markers were allocated on each chromosome using our serial MAALs (Kang et al., 2014). Among those for MAAL Me, 21 SSR markers and 1 centromeric marker showed polymorphisms between the maintainer, woad, MAAL Me, RF 39, F_1_, and *inap* CMS. Their DNA bands were amplified only in woad and MAAL Me (Figure 4A,B), also revealing no existence of intact chromosome e in RF 39. From AFLP analysis with 256 primer combinations, only EA6/MC13, EA8/MC12, EA15/MC6, and EA15/MC12 amplified polymorphic bands in woad, MAAL Me, RF 39, and F_1_ but not in the maintainer and *inap* CMS (Figure 4C), showing the DNA introgression from *I. indigotica* into the genome of RF 39. Unfortunately, we failed to convert these four markers into SCAR markers. The majority of the consensus bands shared by the maintainer, RF 39, and *inap* CMS demonstrated the high similarities of their genomic constitutions.

### Genetic Control of Fertility Restoration

In one F_2_ population with 820 plants derived from the *inap* CMS × RF 39 F_1_ hybrid, which was male fertile, the 3:1 segregation ratio was presented by the male fertile plants (619) and male sterile plants (201) (χ^2^ = 0.08, *p* < 0.01), indicating that the *Rf* gene of RF 39 was a single dominant gene. Furthermore, all the plants in this F_2_ population, which were male fertile, had a brown pollen color, demonstrating that the gene(s) controlling brown pollen color was closely linked to the *Rf* gene of RF 39.

### Genetic Improvement of Restorer RF 39

As the restorer RF 39 exhibited some limitations, such as the less plump anthers with fewer pollen grains, lower seed fertility than the maintainer and the increased GSL content, it was pollinated by 30 rapeseed cultivars of different origins, and their hybrids and selfed progenies were selected for pollen and seed fertility, and for double-low seed qualities and agronomic traits. Three lines were identified to produce plumper anthers with abundant pollen grains and to show the seed sets comparable to the maintainer, as well as the reduced GSL content (<30 μmol/g).

## Discussion

As CMS can be associated with disease susceptibility, the diversification of cytoplasmic origin is beneficial for crop breeding and is more pressing for rapeseed in China, as no other efficient CMS systems other than the predominant *polima* CMS and *shan 2A* CMS, both with CMS gene *orf224*, have been developed in about 30 years. The *polima* CMS has been replaced by the “Ogu-INRA” CMS system and other systems in Europe and Canada (Friedt et al., 2018), while the *M. arvensis*-based CMS and fertility restoration system have also been widely adopted for *B. juncea* in India (Singh, 2008). From these two successful models of development of synthetic alloplasmic CMS and restorers, it is crucial to realize the introgression of *Rf* gene(s) via MAAL as the breeding bridge, where the additional chromosome carries the *Rf* gene(s), by selecting the target plants resulting from the homoeologous recombination or chromosomal translocation (Prakash et al., 1998; present study). In spite of the very distant relationship between rapeseed and Chinese woad from two tribes, the genetic introgression from the additional woad chromosome of MAAL Me into the rapeseed genome was obviously ascertained from the selection of the restorer line, which was also coupled with the woad trait of brown pollen grains. The presence of sterility-inducing cytoplasm in the fertile MAAL should help us to identify the target plants, as those without the introgressed *RF* segment were sterile (Prakash et al., 1998) and were easily identified by their carpelloid stamens in the present study. The frequency of introgression with the *Rf* gene seemed to be very low, as only a few plants with good fertility restoration were identified among a large progeny population.

Even after the successful development of primary restorers for alloplasmic CMS, the male or female fertilities of these restorer lines and other negative traits need further improvements to satisfy the production of commercial hybrids, due to the genetic linkage on the introgression region or the disturbance of genomic structure by the integration of an alien element. Although a single radish nuclear gene, *Rfo*, restored *Ogura* CMS in *B. napus* by altering the translational expression of *orf138* (Bellaoui et al., 1999), the large size of the radish introgression of at least 50 cM carrying the *Rfo* locus led to a poor restorer with deleterious characters, including an increased GSL content (Delourme et al., 1991, 1995). Together with extensive backcrossing and pedigree breeding, molecular markers closely linked to the *Rfo* gene assisted with the selection of the restorer lines with shorter radish fragments and better agronomic performance (Delourme et al., 1991, 1995, 1998; Giancola et al., 2003; Primard-Brisset et al., 2005). In particular, a low GSL restorer line, R2000, which was developed through gamma ray irradiation, deleted radish introgression but recovered some *B. oleracea* sequences that were originally replaced by the introgression (Primard-Brisset et al., 2005; Hu et al., 2008). After the rectification of chlorosis in CMS (*M. arvensis*) *B. juncea*, genetic improvements were made for the parents in 1999–2005, before the release of the hybrids, including the elite NRCHB506 (Singh, 2008; Lei et al., 2018).

Of interest, the increased GSL content also appeared in our restorer for *inap* CMS, compared with the maintainer (Table 2), as observed in the restorer of *Ogura* CMS *B. napus* (Delourme et al., 1995; Primard-Brisset et al., 2005). Although the major GSL component SIN in woad was absent in the restorer RF 39, the extensive increase in NAP from 1.22 μmol/g in the maintainer to 34.15 μmol/g in RF 39 was most likely caused by the genetic introgression or the following interaction, as the woad showed a higher NAP content (9.94 μmol/g). The increased content of PRO from 0.9 μmol/g in the maintainer to 9.56 μmol/g in RF 39 likely resulted from the alien genetic element, as the woad presented the highest PRO content (13.41 μmol/g). The changed GSL profile of RF 39 was probably due to the disturbance of complex GSL biosynthesis, transport, and degradation pathways by alien introgression (Lu et al., 2018). The close association between fertility restoration and the GSL content in the radish introgression was quite difficult to break, while the woad introgression resulted in a looser association that could be more easily modified for the reduced GSL content. This difference was likely not only related to the fact that the *B. napus* donor and the alien introgression restorer did not produce the major GSL type of SIN in the woad but also suggested that the *RF* gene(s) in these two cases were linked with different genes controlling GSL synthesis. This further reflected the structural difference in the genomes of radish and woad, as woad has a much simpler and smaller genome (Lysak and Koch, 2011) than radish (Kitashiba et al., 2014; Jeong et al., 2016), and its genome is about two times greater than that of *Arabidopsis thaliana* (Lysak et al., 2009) or nearly one half of that of *Brassica* diploids. The genomic sequences of the radish and *B. rapa* showed that no significant difference existed in the gene contents related to GSL synthesis (Jeong et al., 2016), except that the radish had fewer myrosinase and thioglucoside glucohydrolase1 genes in the GSL breakdown pathway (Jeong et al., 2016). Specifically, the radish genome had intermediate characteristics between the *Brassica* A/C and B genomes in the triplicated segments, and its nine chromosomes were rearranged from the chromosomes of the hexaploid progenitor, suggesting an internal origin from the genus *Brassica* (Jeong et al., 2016). However, the Isatideae tribe had a rearranged proto-Calepine karyotype (PCK, *n* = 7) with a single copy of all 24 blocks derived from an ancestral cruciferous karyotype (ACK, *n* = 8) (Lysak and Koch, 2011). Since the genetic mapping of the *RF* gene for our restorer line and the development of the linked molecular markers lagged behind, it is now impossible to figure out the genomic region of the *RF* locus and the size of the woad fragment introduced. The ongoing genome sequencing of the Chinese woad genotype used in the present study will provide new opportunities to unravel the genetic structure of the restorer harboring the certain alien fragment and to elucidate the reasons for the linkage with the glucosinolate content, as well as the poor anther dehiscence and brown pollen color.

## Conclusion

In conclusion, the restorer for *inap* CMS of rapeseed was capable of restoring its male fertility by converting the carpelloid stamens into normal stamens with viable pollen grains. The alien introgression of *RF* gene was visibly accompanied by the expression of the brown pollen trait specific to one woad chromosome. The restorer was improved for seed fertility, low glucosinolate content. Thus, a new alloplasmic CMS/fertility restoration system was established for hybrid seed production in *B. napus*, after *Ogura* CMS system in *B. napus* and *Mor* CMS system in *B. juncea*.

## Ethics Statement

The authors declare that the experiments complied with current laws of the country in which they were performed.

## Author Contributions

ZL and LK conceived the experiments. PL performed the research. AW, SG, and XG contributed to phenotypic measurements. CC, LJ, and ZL contributed to restorer improvement. PL and ZL wrote the manuscript. All authors reviewed and approved the final version of the manuscript.

## Conflict of Interest Statement

The authors declare that the research was conducted in the absence of any commercial or financial relationships that could be construed as a potential conflict of interest.
